# Efficacy of emicizumab is maintained throughout dosing intervals for bleed prophylaxis

**DOI:** 10.1016/j.rpth.2023.100077

**Published:** 2023-02-08

**Authors:** Steven W. Pipe, Ben Trzaskoma, Miranda Minhas, Michaela Lehle, Richard H. Ko, Ling Gao, Johnny Mahlangu, Christine L. Kempton, Craig M. Kessler, Rebecca Kruse-Jarres

**Affiliations:** 1 Departments of Pediatrics and Pathology, University of Michigan, Ann Arbor, Michigan, USA; 2 US Medical Affairs, Genentech Inc, South San Francisco, California, USA; 3 Product Development, F. Hoffmann-La Roche Ltd, Basel, Switzerland; 4 Analystat Corporation, Point Roberts, Washington, USA; 5 Department of Molecular Medicine and Haematology, School of Pathology, Faculty of Health Sciences, University of the Witwatersrand and National Health Laboratory Service, Johannesburg, South Africa; 6 Department of Hematology and Medical Oncology and Hemophilia of Georgia Center for Bleeding & Clotting Disorders of Emory, Emory University School of Medicine, Atlanta, Georgia, USA; 7 The Division of Coagulation, Georgetown University School of Medicine, Washington, DC, USA; 8 Division of Hematology, University of Washington and Washington Center for Bleeding Disorders, Seattle, Washington, USA

**Keywords:** bispecific antibody, drug administration schedule, emicizumab, hemophilia A, prophylaxis

## Abstract

**Background:**

Across the HAVEN clinical trial program, the efficacy of emicizumab has been demonstrated in children, adolescents, and adults with hemophilia A, with or without factor VIII inhibitors. After the 4-week loading dose period, emicizumab concentrations are expected to remain at levels that provide bleed protection throughout the entire dosing interval, regardless of the chosen maintenance dosing regimen, ie, weekly, every 2 weeks, or every 4 weeks.

**Objectives:**

The objective of this study was to examine the timing of treated bleeds within the dosing intervals for emicizumab administered during the HAVEN 1 to 4 studies.

**Methods:**

In this post hoc analysis, we pooled data from all the participants of the HAVEN 1 to 4 studies and analyzed the timing of treated bleeds in relation to the emicizumab dose.

**Results:**

A total of 392 participants were included in this analysis, with a median (range) age of 28.0 years (1.1-77.0 years). Target joints were identified in 237 of 392 (60.5%) participants before the study entry. Overall, 211 of 392 (53.8%) participants experienced 907 treated bleeding events. The total mean (SD) annualized bleeding rate across the 4 studies was 1.6 (5.9). There was no evidence that bleeding events clustered on any 1 particular day in any dosing schedule from HAVEN 1 to 4 (*P* > .05 for all 3 treatment regimens).

**Conclusion:**

Data from the HAVEN 1 to 4 trials show consistent bleed prevention within the dosing interval, regardless of the dosing regimen chosen. These findings provide further evidence of the sustained efficacy of emicizumab across all approved dosing regimens to reduce bleeding in people with hemophilia A.

## Essentials

• Emicizumab is approved for subcutaneous prophylaxis weekly, every 2 weeks, or every 4 weeks.
• We analyzed the number and timing of treated bleeds in 392 participants from HAVEN 1 to 4.
• Bleeds were not clustered on any 1 particular day in any of the 3 dosing schedules.
• Analysis demonstrated consistent bleed prevention, regardless of the dosing regimen used.

## 1 Introduction

People with hemophilia A have an X-linked inherited bleeding disorder characterized by a deficiency in coagulation factor (F)VIII, leading to frequent spontaneous and traumatic bleeding, typically into the joints, muscles, and soft tissues [1]. Recurrent bleeds, particularly into the joints, leads to long-term complications, such as debilitating hemophilic arthropathy and an overall reduced quality of life [[1], [2], [3]].

For those with severe hemophilia A (circulating FVIII level of <1 IU/dL) [4], prophylaxis with clotting factor concentrates or hemostatic products, such as emicizumab, is recommended to prevent bleeding [1]. However, a severe bleeding phenotype rather than factor levels is becoming increasingly recognized as an indication for prophylactic treatment [1] because those with baseline endogenous FVIII levels associated with mild and moderate hemophilia A can still experience joint bleeds and develop significant arthropathy [5]. Prophylaxis initiation before 3 years of age is advised to help prevent life-threatening bleeding, such as intracranial hemorrhage, and long-term musculoskeletal complications that can occur because of recurrent joint and muscle bleeds over time [1,6,7]. However, early prophylaxis with standard FVIII prophylaxis has been found insufficient to fully prevent joint damage through adolescence in severe hemophilia A [7].

Owing to the short half-lives of all replacement therapies (approximately 10-16 hours), traditional methods for prophylaxis require multiple intravenous FVIII infusions per week to replace FVIII at levels high enough to provide sufficient protection against bleeds [[8], [9], [10]]. People with hemophilia A are more vulnerable to bleeding when FVIII activity drops below a certain level [11]; historically, trough levels of 1 IU/dL have been targeted [9,11,12]. Increasing amounts of data [13], acknowledged by the recent World Federation of Hemophilia guidelines [1], have indicated that patients remain at risk of occasional clinical and subclinical bleeding with trough levels at 1%, and a trough level of 3% to 5% or higher is more effective in protecting against bleeds and long-term joint damage [1,9,13,14]. Higher trough levels, however, require higher doses or more frequent infusions, which increases costs [1] and can be burdensome to individuals with hemophilia A in terms of psychosocial well-being, physical health, and employment. Therefore, strategies that achieve steady-state hemostasis may be more effective in protecting against bleeds over time [6].

Emicizumab is a subcutaneously administered, bispecific, humanized monoclonal antibody that substitutes for missing or defective FVIII by bridging activated FIX and FX to allow the coagulation cascade to continue in people with hemophilia A [1,15,16]. It can be administered in any of the following 3 dosing regimens: 1.5 mg/kg once weekly, 3 mg/kg every 2 weeks, or 6 mg/kg every 4 weeks. Emicizumab has a long half-life of approximately 4 to 5 weeks [16] and persists in circulation for many months [17]. Plasma emicizumab trough concentrations of approximately 30 μg/mL are maintained whether administered once weekly, every 2 weeks, or every 4 weeks, providing steady-state hemostasis [[18], [19], [20], [21], [22]]. This analysis delves further into the pharmacodynamic efficacy provided by emicizumab to investigate whether there is evidence of waning protection at any given time during the dosing period for any of the 3 approved dosing regimens.

## 2 Methods

### 2.1 Study design and study participants

Data from the HAVEN 1 to 4 Phase III, open-label, multicenter studies (NCT02622321, NCT02795767, NCT02847637, and NCT03020160) were pooled for this retrospective, post hoc analysis (data cutoff: May 15, 2020). An overview of emicizumab dosing cohorts used in HAVEN 1 to 4 is provided in Table 1. The demographics and inclusion and exclusion criteria for all HAVEN 1 to 4 studies have been detailed previously [[18], [19], [20], [21]]. Participants were defined as having FVIII inhibitors if they had inhibitor titers of ≥0.6 Bethesda units/mL, as measured by the chromogenic Bethesda assay.

**Table 1:** Study design overview for emicizumab dosing cohorts in HAVEN 1 to 4.

Clinical trial name and population information	1.5 mg/kg once wk^a^	3 mg/kg every 2 wk^a^	6 mg/kg every 4 wk^a^
HAVEN 1	n = 112		
Adult/adolescent (≥12 y) participants with hemophilia A with FVIII inhibitors			
HAVEN 2	n = 68	n = 10	n = 10
Pediatric^b^ (<12 y) participants with hemophilia A with FVIII inhibitors			
HAVEN 3	n = 99	n = 52	
Adult/adolescent (≥12 y) participants with hemophilia A without FVIII inhibitors			
HAVEN 4			n = 41
Adult/adolescent (≥12 y) participants with hemophilia A^c^ with or without FVIII inhibitors			

FVIII, factor VIII.

a All maintenance doses were preceded by weekly loading doses of 3 mg/kg for 4 weeks.

b Adolescents aged 12 to 17 years were also eligible to enroll in HAVEN 2 if they weighed less than 40 kg.

c Five participants had FVIII inhibitors, and 36 participants did not have FVIII inhibitors at study entry. Two participants included in HAVEN 3, and noted as without FVIII inhibitors in this analysis, had negative results on the inhibitor assay conducted locally to determine eligibility, but inhibitors were detected in centrally tested baseline samples (3.7 and 3.1 Bethesda units/mL, respectively). Titers declined spontaneously during the trial for both participants.

### 2.2 Ethical considerations

All adult participants provided written informed consent before entering the HAVEN studies. Adolescent participants (12-17 years of age) provided written informed assent for the HAVEN studies and children aged 8 to 11 years also provided informed assent for HAVEN 2. Written informed consent was also obtained from parents or legally acceptable representatives in HAVEN 2. All study protocols were approved by the independent ethics committee or institutional review board at each participating institution and were conducted in accordance with the principles of the Declaration of Helsinki and Good Clinical Practice.

### 2.3 Outcomes and data collection

The primary focus of this analysis was the timing of treated bleeding events within the dosing intervals for emicizumab, which were analyzed as the number and percentage of occurrences during different time intervals since the last dose. A treated bleed was defined as a bleed followed by treatment that promotes hemostasis; bleeds due to surgery or procedures were excluded [23]. Two bleeds of the same type (eg, joint, muscle, or other) and at the same anatomical location were considered 1 bleed if the second occurred within 72 hours of the treatment for the first bleed. The timing of the treated bleeding events that occurred during the 4-week loading dose phase among participants in HAVEN 1 to 4, regardless of the inhibitor status, was also analyzed.

The number of treated bleeds associated with previously missed doses of emicizumab was also captured for all 3 regimens. Missed doses are defined as the following: more than 10 days between 2 doses for the weekly regimen (4-day window), more than 21 days between doses for the “every 2 weeks” regimen (7-day window), and more than 42 days between doses for the “every 4 weeks” regimen (14-day window).

Data were collected by using the same methodology across all 4 studies; bleeds and treatment were self-reported by using the Bleed and Medication Questionnaire. Participants with hemophilia A receiving emicizumab by either treatment assignment or switch to emicizumab following the initial assignment of no prophylaxis were included in the analysis. For participants with changes to their dose, only data from the original dosing regimen were included.

### 2.4 Data analysis

Bleed analyses were summarized for the entire dosing period, including the loading (3 mg/kg once weekly for 4 weeks) and maintenance phases (1.5 mg/kg once weekly, 3 mg/kg every 2 weeks, or 6 mg/kg every 4 weeks). Annualized bleed rates (ABR; both overall treated and treated traumatic bleed rates) were calculated at the patient level by dividing the individual observed bleeds by the respective exposure time, as a fraction of a year. Taking the average of these numbers across all participants produced the mean ABR and taking the median of these numbers produced the median ABR. Each treated bleed was categorized as having occurred on a given day of the participant’s dosing interval from the last emicizumab dose (eg, days 1-7 for those treated weekly, day 1-14 for those treated every 2 weeks, and days 1-28 for those treated every 4 weeks). For any analyses related to the day in which a bleed occurred, including those in the subgroup analyses, the percentage of the bleeds falling on a given day was calculated and summarized.

Sensitivity analyses of the bleeding rates in the individual studies found no discernible differences between the studies; accordingly, adjustments to the pooled analysis on the basis of inter-study differences were not warranted. Similarly, no substantial differences in the patterns of bleeding were observed in a sensitivity analysis between those on emicizumab at the outset and those who switched to emicizumab following an initial assignment of no prophylaxis.

Analysis of the association between the occurrence of bleeds and the day in which a bleed occurred relative to the most recent dose of emicizumab was performed by using a generalized linear model procedure (PROC GENMOD with a repeated statement, negative binomial distribution, and log link), with time as a factor. *P* values <.05 were considered statistically significant. All analyses were conducted by using SAS v9.4 software.

## 3 Results

### 3.1 Baseline participant and treatment characteristics

In total, 401 participants were enrolled in HAVEN 1 to 4 (Supplementary Fig. 1). One participant in HAVEN 1 discontinued before emicizumab treatment and was excluded from this analysis. Another participant discontinued on day 24 of emicizumab because of the physician’s decision and is therefore only included in the loading dose analysis. One participant in HAVEN 3 was assigned to no prophylaxis but was lost to follow up and received no emicizumab treatment; this participant was excluded from all analyses on this basis. Only the HAVEN 4 expansion cohort (n = 41) was included in this analysis; the run-in cohort (n = 7) was excluded because of the use of a different dosing regimen (6 mg/kg every 4 weeks for 24 weeks without a loading dose).

Overall, 392 participants across HAVEN 1 to 4 were included in the present analysis (Table 2). The median age (range) across all HAVEN studies was 28.0 years (1.1-77.0). The median (interquartile range [IQR]) duration of exposure to emicizumab for all 4 studies was 120.1 (88.9-164.2) weeks. Across HAVEN 1 to 4, 237 of 392 participants (60.5%) had target joints before the study entry and 205 of 392 (52.3%) had inhibitors against FVIII.

**Table 2:** Baseline participant characteristics by emicizumab dosing regimen (HAVEN 1-4 pooled).

Emicizumab maintenance dosing regimen	1.5 mg/kg once wk				3 mg/kg every 2 wk			6 mg/kg every 4 wk			Total^a^
	HAVEN 1	HAVEN 2	HAVEN 3	Total	HAVEN 2	HAVEN 3	Total	HAVEN 2	HAVEN 4	Total	
Cohorts, n	112	68	99	279	10	52	62	10	41	51	392
Age (y), median (range)	28.5 (12.0-75.0)	6.8 (1.1-15.9)	36.0 (13.0-77.0)	26.0 (1.1-77.0)	8.3 (2.6-11.0)	41.0 (16.0-65.0)	38.0 (2.6-65.0)	9.60 (2.1-11.6)	39.0 (14.0-68.0)	29.0 (2.1-68.0)	28.0 (1.1-77.0)
Race, n (%)											
American Indian or Alaska Native	1 (0.9)	0 (0)	0 (0)	1 (0.4)	0 (0)	0 (0)	0 (0)	0 (0)	0 (0)	0 (0)	1 (0.3)
Asian	21 (18.8)	10 (14.7)	18 (18.2)	49 (17.6)	1 (10.0)	13 (25.0)	14 (22.6)	2 (20.0)	8 (19.5)	10 (19.6)	73 (18.6)
Black or African American	11 (9.8)	11 (16.2)	4 (4.0)	26 (9.3)	1 (10.0)	4 (7.7)	5 (8.1)	0 (0)	1 (2.4)	1 (2.0)	32 (8.2)
Native Hawaiian or other Pacific Islander	1 (0.9)	0 (0)	1 (1.0)	2 (0.7)	0 (0)	0 (0)	0 (0)	0 (0)	0 (0)	0 (0)	2 (0.5)
White	74 (66.1)	39 (57.4)	71 (71.7)	184 (66.0)	7 (70.0)	31 (59.6)	38 (61.3)	8 (80.0)	31 (75.6)	39 (76.5)	261 (66.6)
Multiple	0 (0)	2 (2.9)	0 (0)	2 (0.7)	0 (0)	0 (0)	0 (0)	0 (0)	0 (0)	0 (0)	2 (0.5)
Unknown	4 (3.6)	6 (8.8)	5 (5.1)	15 (5.4)	1 (10.0)	4 (7.7)	5 (8.1)	0 (0)	1 (2.4)	1 (2.0)	21 (5.4)
Ethnicity, n (%)											
Hispanic or Latino	18 (16.1)	5 (7.4)	7 (7.1)	30 (10.8)	1 (10.0)	4 (7.7)	5 (8.1)	1 (10.0)	2 (4.9)	3 (5.9)	38 (9.7)
Not Hispanic or Latino	94 (83.9)	61 (89.7)	92 (92.9)	247 (88.5)	9 (90.0)	47 (90.4)	56 (90.3)	9 (90.0)	38 (92.7)	47 (92.2)	350 (89.3)
Not reported or unknown	0 (0)	2 (2.9)	0 (0)	2 (0.7)	0 (0)	1 (1.9)	1 (1.6)	0 (0)	1 (2.4)	1 (2.0)	4 (1.0)
Duration of exposure^b^ (wk), median (IQR)	109.8 (92.1-167.7)	96.3 (74.7-138.6)	163.6 (108.1-172.1)	114.4 (92.0-166.1)	67.14 (66.1-122.1)	154.1 (108.1-169.1)	141.3 (96.6-166.6)	66.6 (32.0-121.0)	150.1 (84.1-152.3)	120.1 (77.4-152.1)	120.1 (88.9-164.2)
Total patient years of emicizumab exposure	262.2	133.4	265.0	660.6	16.82	138.8	155.6	14.3	91.5	105.8	922.0
FVIII inhibitors, n (%)											
Yes	112 (100.0)	68 (100.0)	-	180 (64.5)	10 (100.0)	-	10 (16.1)	10 (100.0)	5 (12.2)	15 (29.4)	205 (52.3)
No	-	-	99 (100.0)	99 (35.5)	-	52^c^ (100.0)	52 (83.9)	-	36 (87.8)	36 (70.6)	187 (47.7)
Presence of target joints, n (%)	77 (69.4)	24 (35.3)	60 (60.6)	161 (57.9)	7 (70.0)	41 (78.9)	48 (77.4)	3 (30.0)	25 (61.0)	28 (54.9)	237 (60.5)
Time from FVIII inhibitor diagnosis date (y), mean (SD)	21.4 (12.7)	4.8 (3.3)	NE	15.0 (13.0)	5.9 (2.9)	NE	5.9 (2.9)	5.8 (3.2)	19.6 (10.7)	13.0 (10.6)	14.4 (12.6)

FVIII, factor VIII; IQR, interquartile range; NE, not evaluable.

a One participant in HAVEN 1 discontinued prior to emicizumab treatment and was excluded; another participant discontinued on day 24 of emicizumab due to the physician’s decision and is therefore only included in the loading dose analysis. One participant in HAVEN 3 was assigned to no prophylaxis but was lost to follow up and was therefore excluded from all analyses on this basis. Only the HAVEN 4 expansion cohort (n = 41) was included in this analysis; the run-in cohort (n = 7) was excluded due to the use of a different dosing regimen (6 mg/kg once weekly for 24 weeks with no loading dose).

b Data represent emicizumab exposure during the efficacy period only.

c Two participants included in HAVEN 3 and noted as without FVIII inhibitors in this analysis had negative results on the inhibitor assay conducted locally to determine eligibility, but inhibitors were detected in centrally tested baseline samples (3.7 and 3.1 Bethesda units/mL, respectively). Titers declined spontaneously during the trial for both participants.

### 3.2 Timing of treated bleeds

**Figure 1:**
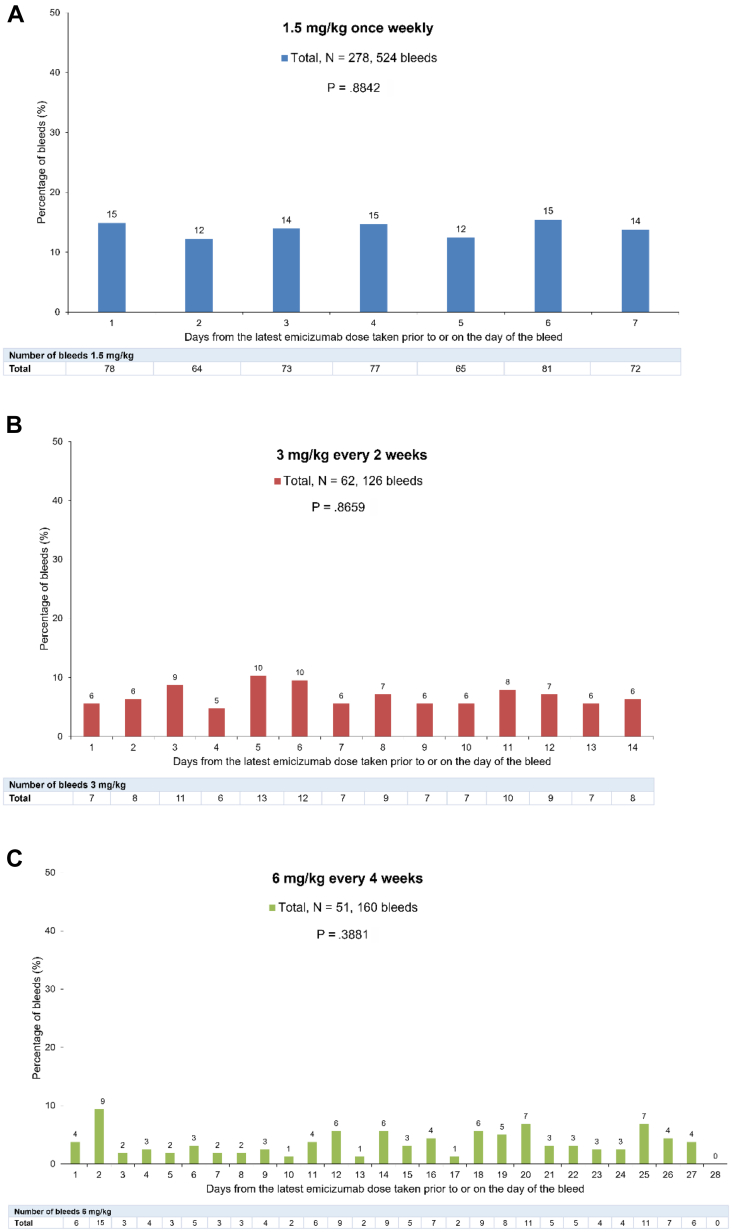
Proportion of treated bleeds occurring at different times since the latest dose of emicizumab in the participants of HAVEN 1 to 4 during the maintenance treatment phase. The total percentage of bleeds by days from the most recent dose was calculated within each study (HAVEN 1-4). Bleeds that occurred past the intended dosing interval window are not shown on the bar chart; this includes 14 bleeds for the weekly regimen, 5 bleeds for the “every 2 weeks” regimen, and 2 bleeds for the “every 4 weeks” regimen. A sensitivity analysis identified no significant association between the 3 emicizumab regimens and bleeding patterns observed (*P* = .8624).

**Figure 2:**
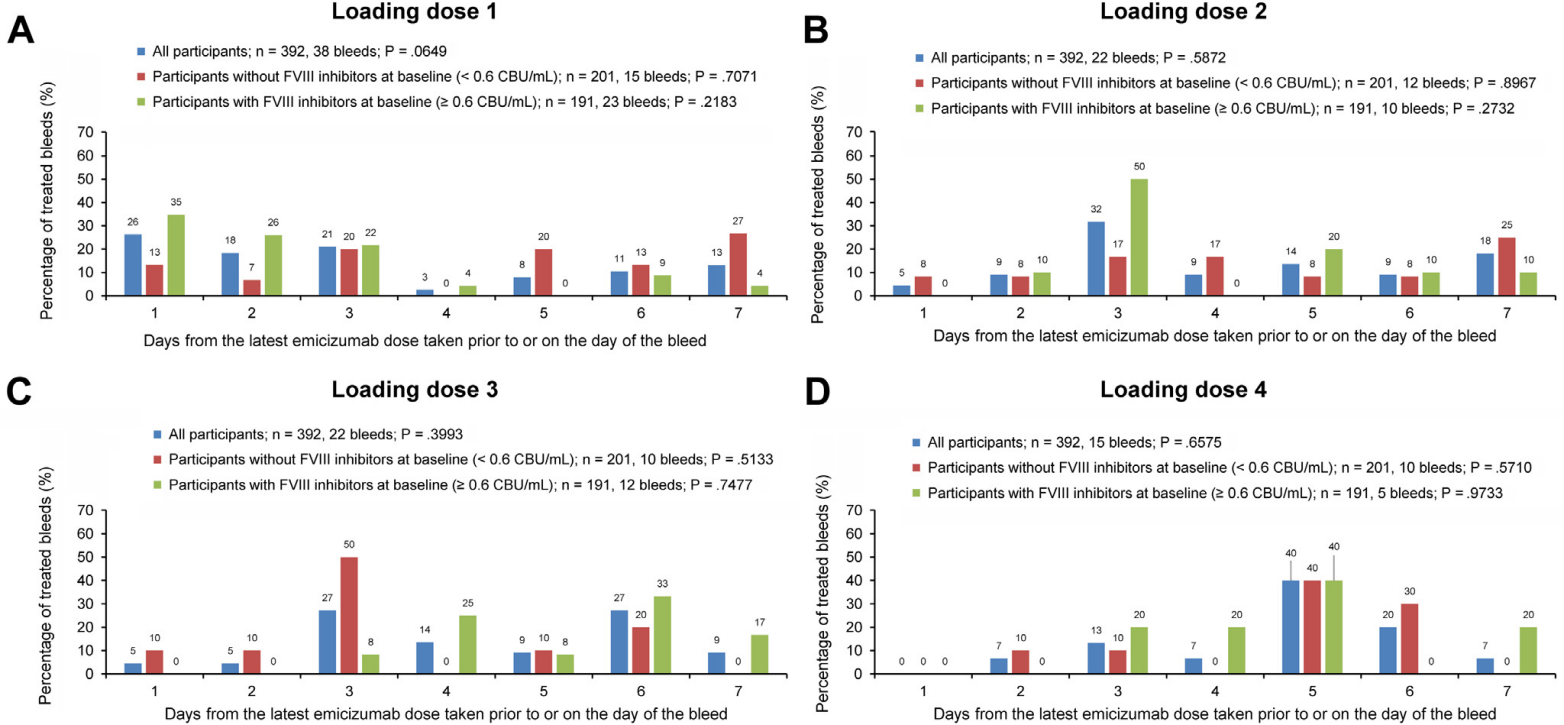
Proportion of treated bleeds occurring at different times since the latest dose of emicizumab in the participants of HAVEN 1 to 4 during the loading dose phase (3 mg/kg once weekly). Days from the latest emicizumab dose may cross between weeks. Bleeds that occurred past the intended dosing interval window are not shown on the bar chart; this includes 1 bleed on day 9 of loading dose 2, 1 bleed on day 8 of loading dose 3, and 1 bleed on day 9 of loading dose 4. CBU, chromogenic Bethesda unit; FVIII, factor VIII.

In the HAVEN 1–4 pooled analysis, 211 of 392 participants (53.8%) experienced a total of 907 treated bleeding events across the loading and maintenance dose periods (Table 3 and Supplementary Table). The total mean (SD) ABR for treated bleeds across the 4 studies, with a median (IQR) duration of exposure to emicizumab of 120.1 (88.9-164.2) weeks, was 1.6 (5.9) and the median ABR (IQR) was 0.3 (0.0-1.0). The overall mean (SD) ABR for the 1.5 mg/kg once weekly, 3 mg/kg every 2 weeks, and 6 mg/kg every 4 weeks dosing regimens was 1.7 (6.7), 0.9 (1.5), and 2.1 (3.9), respectively, with a median (IQR) ABR of 0.3 (0.0-1.0), 0.3 (0.0-1.0), and 0.6 (0.0-2.4) for each of the 3 dosing regimens, respectively. Of the 907 treated bleeding events, 588 (64.8%) were traumatic. There was no evidence of an uneven distribution of treated bleeds across different days in any dosing schedule from HAVEN 1 to 4 (Figure 1; *P* > .05 for all 3 treatment regimens). Furthermore, a sensitivity analysis identified no significant association between the 3 emicizumab regimens and bleeding patterns observed (*P* = .8624). An equal distribution of treated bleeds across different days in the initial 4-week loading dose period was observed, with no difference for those with or without inhibitors against FVIII (*P* > .05 for all patient groups; Figure 2). The number of bleeds declined across weeks 1 to 4 of the loading dose phase for those with and without FVIII inhibitors.

**Table 3:** Number of treated bleeds across the whole treatment period.

Emicizumab maintenance dosing regimen	1.5 mg/kg once wk				3 mg/kg every 2 wk			6 mg/kg every 4 wk			Total	
	HAVEN 1	HAVEN 2	HAVEN 3	Total	HAVEN 2	HAVEN 3	Total	HAVEN 2	HAVEN 4	Total		
Study cohort, n	112	68	99	279	10	52	62	10	41	51	392	
Participants with treated bleeds, n (%) No. of bleeds	55 (49.1) 261	24 (35.3) 46	65 (65.7) 290	144 (51.6) 597	3 (30.0) 3	31 (59.6) 136	34 (54.8) 139	4 (40.0) 8	29 (70.7) 163	33 (64.7) 171	211 (53.8) 907	
Mean ABR for treated bleeds (SD)	2.6 (10.2)	0.3 (0.6)	1.5 (2.8)	1.7 (6.7)	0.2 (0.3)	1.0 (1.6)	0.9 (1.5)	2.2 (4.0)	2.1 (4.0)	2.1 (3.9)	1.6 (5.9)	
Median ABR for treated bleeds (IQR)	0.0 (0.0-1.1)	0.0 (0.0-0.6)	0.5 (0.0-1.3)	0.3 (0.0-1.0)	0.0 (0.0-0.4)	0.3 (0.0-1.5)	0.3 (0.0-1.0)	0.0 (0.0-3.3)	0.6 (0.0-1.8)	0.6 (0.0-2.4)	0.3 (0.0-1.0)	
Participants with treated traumatic bleeds, n (%) No. of bleeds	38 (33.9) 136	23 (33.8) 38	47 (47.5) 177	108 (38.7) 351	3 (30.0) 3	28 (53.9) 97	31 (50.0) 100	3 (30.0) 4	23 (56.1) 133	26 (51.0) 137	165 (42.1) 588	
Mean ABR for treated traumatic bleeds (SD)	1.2 (4.5)	0.3 (0.5)	0.8 (2.0)	0.9 (3.1)	0.2 (0.3)	0.7 (1.3)	0.6 (1.2)	1.6 (3.7)	1.5 (3.5)	1.5 (3.5)	0.9 (3.0)	
Median ABR for treated traumatic bleeds (IQR)	0.0 (0.0-0.5)	0.0 (0.0-0.5)	0.0 (0.0-0.9)	0.0 (0.0-0.6)	0.0 (0.0-0.4)	0.3 (0.0-1.0)	0.1 (0.0-0.9)	0.0 (0.0-0.8)	0.3 (0.0-1.4)	0.3 (0.0-1.4)	0.0 (0.0-0.7)	

The whole treatment period includes the loading phase, which was the same for all regimens (3 mg/kg once weekly for 4 weeks) and the maintenance phases (1.5 mg/kg once weekly, 3 mg/kg every 2 weeks, or 6 mg/kg every 4 weeks).

ABR, annualized bleeding rate; IQR, interquartile range.

Adherence to dosing schedules was good throughout all trials. Of an expected 40,307 doses among all 392 participants, 80 participants (20.4%) missed at least 1 dose (159 missed doses in total). Mean (SD) ABRs calculated for the subset with at least 1 missed dose and the subset without any missed doses (n = 312) were 1.3 (3.4) and 1.7 (6.4), respectively. Twenty-eight participants experienced treated bleeding events following a missed dose, with most occurring well after the maintenance dose regimen was resumed (Supplementary Fig. 2).

## 4 Discussion

In this analysis of the timing of treated bleeds across the emicizumab dosing schedules included in HAVEN 1 to 4, no evidence of any association between the occurrence of treated bleeds and the time of last dose of emicizumab was observed.

Similar to the long-term pooled analysis of HAVEN 1 to 4 by Callaghan et al. 2021, no notable differences in bleed rates were observed between dosing regimens [23]. The ABR for treated bleeds remained low for all dosing schedules (total mean ABR 1.6; total median ABR 0.3), with more than 60% of the treated bleeds across the 4 studies being due to trauma. The mean ABR reported here differs slightly from that reported in the long-term pooled data analysis of HAVEN 1 to 4 [23]. The difference in mean ABR may be because, in the current analysis, the total mean ABR (1.6) was derived without adjustment to be consistent with how the percentage of bleeds by dosing interval was derived, whereas in the long-term pooled data analysis of HAVEN 1 to 4, the mean ABR (1.4) was a model-based ABR for the period of the sum of only the 24-week intervals for which the participants completed emicizumab prophylaxis. When using a negative binomial model adjusted by the number of bleeds in the 24 weeks before the study entry, the adjusted ABRs for treated bleeds are slightly lower than the unadjusted ABRs shown in Table 3 and the Supplementary Table (1.17, 2.99, and 1.09 for the whole treatment period, the loading dose period, and the maintenance dose period, respectively). Differences in ABR could also be explained by incomplete patient bleed records (cut before the last dosing period was complete) and the different numbers of participants included in the studies (400 in the long-term pooled analysis vs 392 in this analysis) because of the exclusion of the HAVEN 4 run-in cohort and 1 patient who was lost to follow up. Importantly, there was no evidence that bleeds clustered toward the end of the dosing period. Although for the “every 2 weeks” and “every 4 weeks” regimens, there appear to be high proportions of treated bleeds in the HAVEN 2 cohort at certain time points, this is likely because of the sample size of the cohort being extremely small (n = 10 for each of these groups) (Supplementary Fig. 3). However, little difference was observed in the median ABRs (IQR) for treated bleeds for the 3 mg/kg every 2 weeks regimen in HAVEN 2 (0.0 [0.0-0.4]) and HAVEN 3 (0.3 [0.0-1.5]), as well as for the median ABRs for the 6 mg/kg every 4 weeks regimen in HAVEN 2 (0.0 [0.0-3.3]) and HAVEN 4 (0.6 [0.0-1.8]) [[19], [20], [21]].

The number of bleeds declined from weeks 1 to 4 of the loading dose period; however, there was no apparent trend in the distribution of bleeds within the dosing intervals during each week. Compared with those treated on demand before enrolling into the HAVEN 1 to 4 studies, the participants who were receiving FVIII prophylaxis before entering Arm D of HAVEN 3 or the expansion cohort of HAVEN 4 continued FVIII prophylaxis until the second loading dose of emicizumab, which may confound the data for week 1 of the loading dose phase.

Overall, these findings are consistent with the half-life of emicizumab and the sustained stable plasma concentrations previously reported with once weekly, every 2 weeks, and every 4 weeks maintenance dosing [15,[24], [25], [26]]. Exposure–response modeling has further demonstrated that emicizumab concentrations of approximately 30 μg/mL are predicted to provide clinically meaningful control of bleeding (please refer to figure 4 in the study of Jonsson et al. 2021); these concentrations can be achieved with all approved dosing regimens of emicizumab [26]. In the HAVEN program, the relationship between plasma emicizumab concentration levels and ABR almost reaches a plateau above 30 μg/mL, which may suggest that this concentration achieves the optimal effect in terms of providing protection against bleeds [26].

Across HAVEN 1 to 4, 79.6% of participants did not miss a dose of emicizumab, indicating high overall adherence with the study protocols. Across all dosing regimens, only a minority of treated bleeding events occurred in participants who had a previously missed dose. A previously published descriptive assessment of the relationship between emicizumab exposure and the rate of bleeding events among the clinical trial participants identified a minor trend toward a reduction in the ABR with increasing median emicizumab concentration, suggesting that reducing emicizumab exposure to lower levels may potentially increase the risk of bleeding [22]. Because there was only a small percentage of missed emicizumab doses in this analysis (0.4%, n = 159/40,307), we did not identify any trends between missing a dose and subsequent bleeding events requiring treatment with FVIII or bypassing agents.

The “every 4 weeks” regimen has demonstrated slightly lower mean steady-state trough plasma concentrations in the HAVEN 4 study (∼40 μg/mL) compared with dosing once weekly or every 2 weeks in the HAVEN 1 to 3 studies (∼52 μg/mL or ∼48 μg/mL, respectively); however, emicizumab concentrations were maintained at levels associated with efficacy [20,27]. The choice of regimen can therefore be dependent on individual patient preference, considering desired lifestyle, without the need for pharmacokinetic monitoring [20].

Findings from the preliminary literature reports of the real-world use of emicizumab have shown that, specifically in the United States, the shorter emicizumab dosing regimens are prescribed more frequently than the longer 6 mg/kg every 4 weeks regimen in people with hemophilia A [28,29]. Findings from the present analysis do not give any reason to doubt the efficacy of emicizumab at any point during the dosing interval for 6 mg/kg every 4 weeks, and therefore, the choice of regimen should be dependent on the patient factors and preferences. The decision on which regimen to start with may be influenced by vial size, weight, lifestyle needs, and considerations around product wastage. Although the explicit reasoning behind the reduced utilization of the longer regimen is unknown, it could perhaps be due to the 6 mg/kg every 4 weeks regimen being perceived as less effective. The findings of this study demonstrate no correlation between increased dosing intervals of emicizumab and waning protection against bleeding.

For limitations, because of the inherent bias associated with post hoc analyses, these data should be interpreted with caution. HAVEN 2 had low numbers of participants in the 3 mg/kg every 2 weeks and 6 mg/kg every 4 weeks emicizumab dosing regimen cohorts; this provided a small sample size for comparison against HAVEN 3 and HAVEN 4 for these dosing intervals [[19], [20], [21]]. Furthermore, data from the Phase III STASEY trial were not included in this analysis, and the occurrence and timing of untreated bleeds from the HAVEN 1 to 4 studies were also not considered.

### 5 Conclusion

No trends were observed with regard to the timing of treated bleeds within the dosing intervals of emicizumab either once weekly, every 2 weeks, or every 4 weeks that would indicate waning protection against bleeds. The 3 approved dosing regimens for emicizumab provide people with hemophilia A with options that can match their lifestyle and help them maintain adherence without impacting efficacy.
