# Supplementary material for: Efficacy of emicizumab is maintained throughout dosing intervals for bleed prophylaxis
Source: Res Pract Thromb Haemost. 2023 Feb 8;7(2):100077. doi: 10.1016/j.rpth.2023.100077 (PMC9992752; doi:10.1016/j.rpth.2023.100077)
Supplement: Supplementary Material [file mmc1.docx]

**Supplementary tables and figures**

**SUPPLEMENTARY TABLE S1.** Number of treated bleeds across the loading and maintenance dose periods

| **Emicizumab dosing regimen** | **1.5 mg/kg once weekly** | | | | **3 mg/kg every 2 weeks** | | | **6 mg/kg every 4 weeks** | | | **Total** |
| --- | --- | --- | --- | --- | --- | --- | --- | --- | --- | --- | --- |
| **Loading dose period** | | | | | | | | | | | |
| **Study**  n | **HAVEN 1** n = 112 | **HAVEN 2** n = 68 | **HAVEN 3** n = 99 | **Total**  n = 279 | **HAVEN 2** n = 10 | **HAVEN 3** n = 52 | **Total**  n = 62 | **HAVEN 2** n = 10 | **HAVEN 4** n = 41 | **Total** n = 51 | **N = 392** |
| **Participants with treated bleeds, n (%)**  No. of bleeds | 23 (20.5) 48 | 0 (0.0)  0 | 21 (21.2)  25 | 44 (15.8)  73 | 0 (0.0)  0 | 6 (11.5)  13 | 6 (9.7)  13 | 1 (10.0)  1 | 8 (19.5)  10 | 9 (17.7)  11 | 59 (15.1)  97 |
| **Mean ABR for treated bleeds**  (SD) | 5.6  (13.6) | 0  (0.0) | 3.3  (6.8) | 3.4  (9.7) | 0.0  (0.0) | 3.2  (11.2) | 2.7  (10.3) | 1.3  (4.1) | 3.2  (7.2) | 2.8  (6.7) | 3.2  (9.5) |
| **Median ABR for treated bleeds**  (IQR) | 0.0  (0.0–0.0) | 0.0  (0.0–0.0) | 0.0  (0.0–0.0) | 0.0  (0.0–0.0) | 0.0  (0.0–0.0) | 0.0  (0.0–0.0) | 0.0  (0.0–0.0) | 0.0  (0.0–0.0) | 0.0  (0.0–0.0) | 0.0  (0.0–0.0) | 0.0  (0.0–0.0) |
| **Participants with treated traumatic bleeds, n (%)**  No. of bleeds | 15 (13. 4)  25 | 0 (0)  0 | 12 (12.1)  15 | 27 (9.7)  40 | 0 (0)  0 | 3 (5.8)  9 | 3 (4.8)  9 | 0 (0)  0 | 7 (17.1)  9 | 7 (13.7)  9 | 37 (9.4)  58 |
| **Mean ABR for treated traumatic bleeds**  (SD) | 2.9  (8.5) | 0.0  (0.0) | 2.0  (5.7) | 1.9  (6.5) | 0.0  (0.0) | 2.3  (10.6) | 1.9  (9.7) | 0.0  (0.0) | 2.9  (7.0) | 2.3  (6.4) | 1.9  (7.0) |
| **Median ABR for treated traumatic bleeds**  (IQR) | 0.0  (0.0–0.0) | 0.0  (0.0–0.0) | 0.0  (0.0–0.0) | 0.0  (0.0–0.0) | 0.0  (0.0–0.0) | 0.0  (0.0–0.0) | 0.0  (0.0–0.0) | 0.0  (0.0–0.0) | 0.0  (0.0–0.0) | 0.0  (0.0–0.0) | 0.0  (0.0–0.0) |
| **Maintenance dose period** | | | | | | | | | | | |
| **Study**  n | **HAVEN 1** n = 111 | **HAVEN 2** n = 68 | **HAVEN 3** n = 99 | **Total**  n = 278 | **HAVEN 2** n = 10 | **HAVEN 3** n = 52 | **Total**  n = 62 | **HAVEN 2** n = 10 | **HAVEN 4** n = 41 | **Total** n = 51 | N = 391 |
| **Participants with treated bleeds, n (%)**  No. of bleeds | 51 (46.0) 213 | 24 (35.3)  46 | 62 (62.6)  265 | 137 (49.3)  524 | 3 (30.0)  3 | 30 (57.7)  123 | 33 (53.2)  126 | 4 (40.0)  7 | 28 (68.3)  153 | 32 (62.8)  160 | 202 (51.7)  810 |
| **Mean ABR for treated bleeds**  (SD) | 2.6  (10.7) | 0.3  (0.6) | 1.4  (2.9) | 1.6  (7.0) | 0.2  (0.3) | 1.0  (1.5) | 0.8  (1.4) | 3.2  (6.7) | 2.1  (4.0) | 2.3  (5.0) | 1.6  (6.2) |
| **Median ABR for treated bleeds**  (IQR) | 0.0  (0.0–1.0) | 0.0  (0.0–0.6) | 0.4  (0.0–1.3) | 0.0  (0.0–0.9) | 0.0  (0.0–0.4) | 0.3  (0.0–1.3) | 0.3  (0.0–1.1) | 0.0  (0.0–4.4) | 0.7  (0.0–1.9) | 0.7  (0.0–2.1) | 0.3  (0.0–1.0) |
| **Participants with treated traumatic bleeds, n (%)**  No. of bleeds | 36 (32.4)  111 | 23 (33.8)  38 | 45 (45.5)  162 | 104 (37.4)  311 | 3 (30.0)  3 | 27 (52.0)  88 | 30 (48.4)  91 | 3 (30.0)  4 | 21 (51.2)  124 | 24 (47.1)  128 | 158 (40.4)  530 |
| **Mean ABR for treated traumatic bleeds**  (SD) | 1.1  (4.5) | 0.3  (0.6) | 0.8  (2.0) | 0.8  (3.1) | 0.2  (0.3) | 0.6  (1.1) | 0.6  (1.1) | 2.7  (6.8) | 1.4  (3.5) | 1.7  (4.3) | 0.9  (3.1) |
| **Median ABR for treated traumatic bleeds**  (IQR) | 0.0  (0.0–0.5) | 0.0  (0.0–0.5) | 0.0  (0.0–0.9) | 0.0  (0.0–0.6) | 0.0  (0.0–0.4) | 0.3  (0.0–1.0) | 0.0  (0.0–0.9) | 0.0  (0.0–0.9) | 0.4  (0.0–1.2) | 0.0  (0.0–1.2) | 0.0  (0.0–0.7) |

ABR, annualized bleeding rate; IQR, interquartile range; SD, standard deviation

**SUPPLEMENTARY FIGURE S1.** CONSORT diagram of HAVEN 1–4 pooled analysis


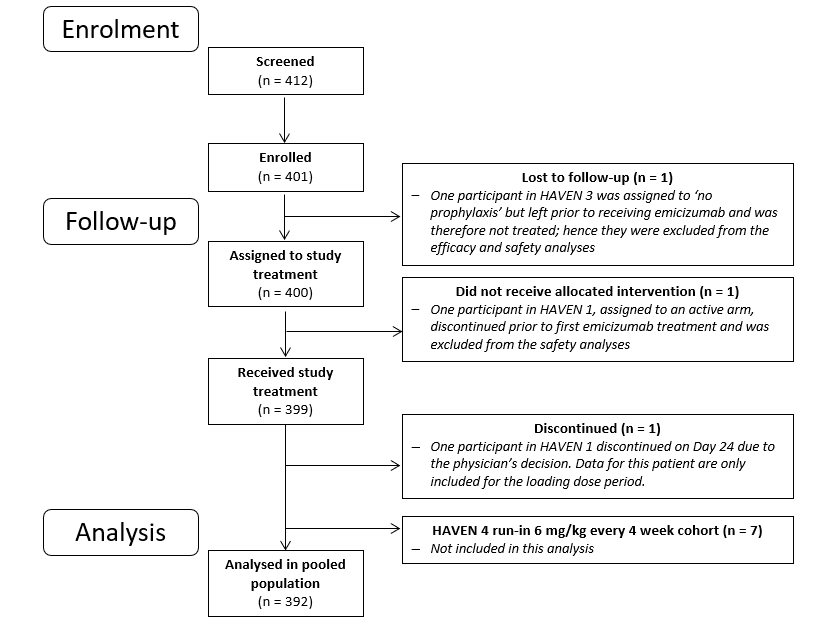


**SUPPLEMENTARY FIGURE S2.** Participants with hemophilia A who had a treated spontaneous or traumatic bleed after missing a dose of emicizumab prophylaxis in the HAVEN 1–4 studies (during the whole treatment period)


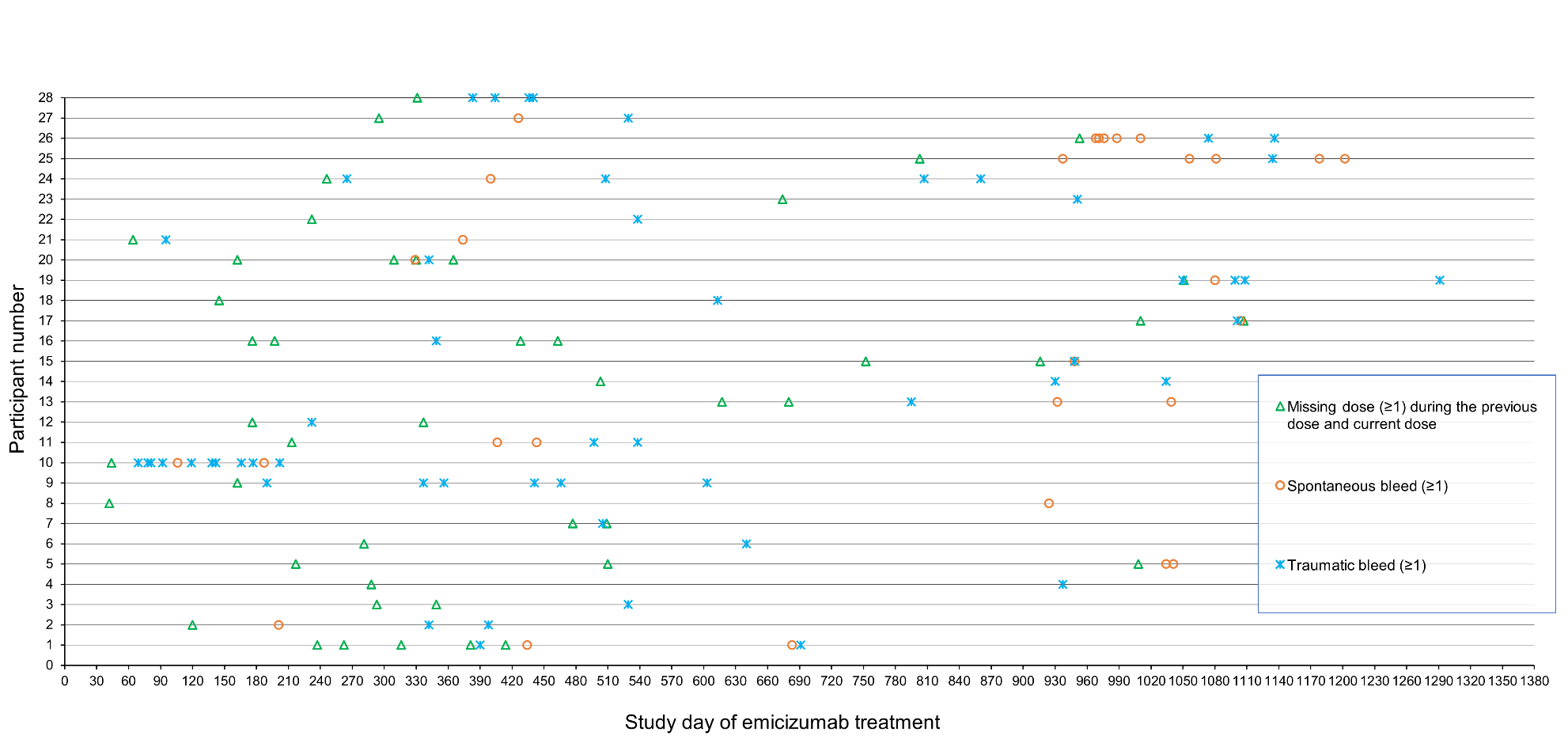


Each point on the chart refers to a patient case. Missed treatments are represented by green triangles, whilst bleeds are represented as spontaneous (orange circle) or traumatic (blue cross). The study day on which the event occurred is shown on the X-axis. The relationship between the bleed and missed dose can be assessed by the relationship of the events across time.

**SUPPLEMENTARY FIGURE S3**. Proportion of treated bleeds occurring at different times since the latest dose of emicizumab in the participants of HAVEN 1–4 during the maintenance treatment phase (broken down by study).


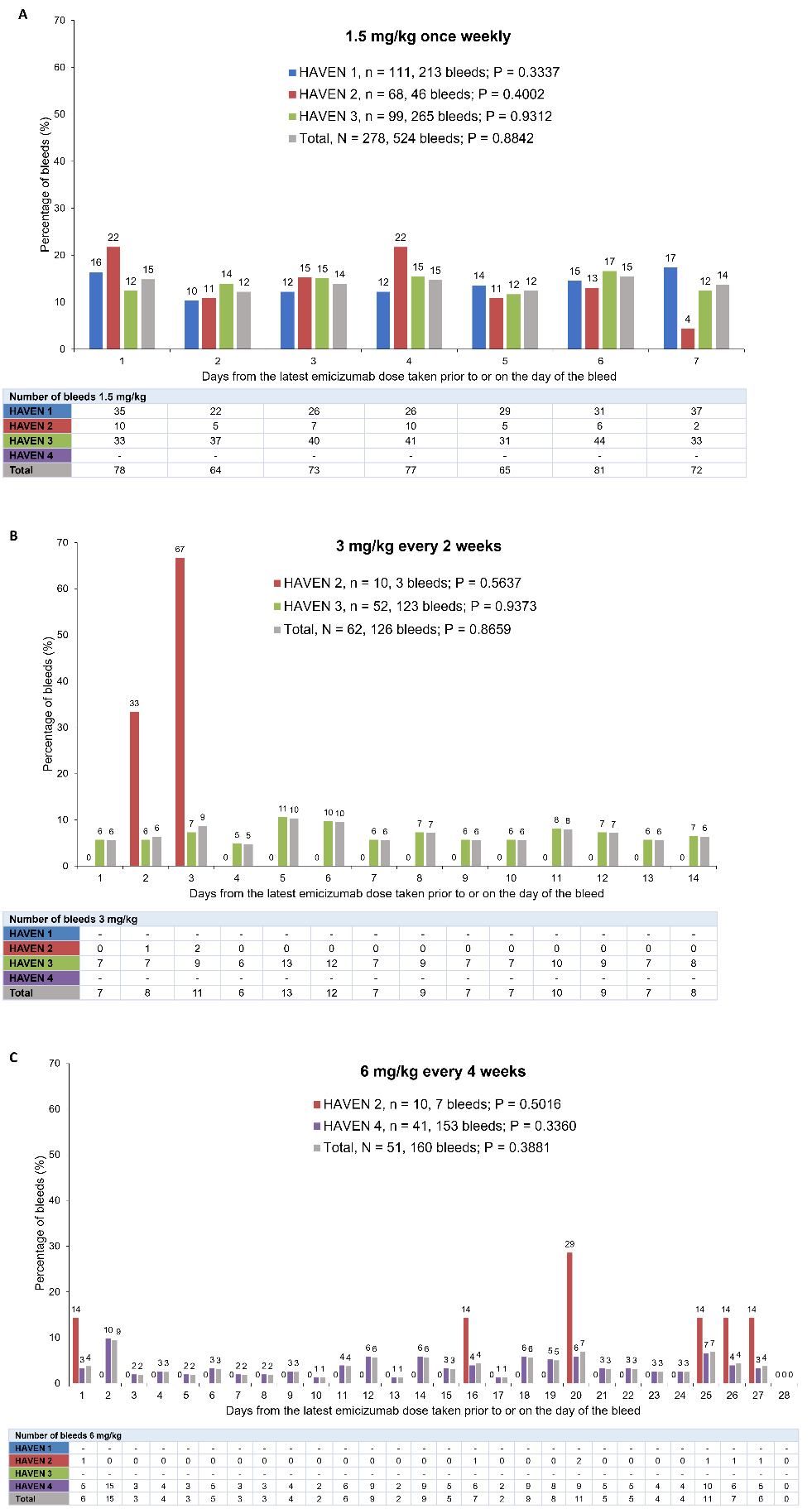


The percentage of bleeds by days from the most recent dose were calculated within each study (HAVEN 1–4). Bleeds that occurred past the intended dosing interval window are not shown on the bar chart; this includes 14 bleeds for the once weekly regimen, five bleeds for the ‘every 2 weeks’ regimen and two bleeds for the ‘every 4 weeks’ regimen.
